# Does internet use promote mental health among middle-aged and older adults in China?

**DOI:** 10.3389/fpsyg.2022.999498

**Published:** 2022-11-15

**Authors:** Chong Zhang, Yan Wang, Jing Wang, Xin Liu

**Affiliations:** ^1^Institute of Network Society Governance, School of Marxism, University of Electronic Science and Technology of China, Chengdu, China; ^2^School of Law and Sociology, XiHua University, Chengdu, China; ^3^Department of Pediatrics, The University of Melbourne, Melbourne, VIC, Australia

**Keywords:** internet use, middle-aged and older adults, mental health, depression, cognition

## Abstract

In recent years, China’s Internet penetration rate has increased, and the scale of middle-aged and older adults’ netizen has continued to expand. However, the impact of internet use on mental health remains controversial. This paper analyzes 14,497 middle-aged and older adults’ valid respondents in the 2018 Chinese Family Panel Study (CFPS2018) to evaluate the impact of Internet use on the mental health of middle-aged and older adults and its intermediary mechanisms. The findings show that moderate use of the Internet can significantly reduce depression levels and boost cognitive function in middle-aged and older adults. But excessive internet use will also lead to increased levels of depression and decreased cognitive function. Different Internet use purposes will also lead to different psychological states. Online socializing, entertainment and business activities can significantly reduce depression levels and promote cognitive functions. Online studying and working only have positive effects on cognitive functions, which have no significant relationship to depression levels. In addition, analysis of the mediation effect found that life satisfaction is a path mechanism for Internet use and affect different dimensions of mental health.

## Introduction

Mental health refers to “a state of well-being in which an individual can recognize his or her abilities, is able to cope with the normal stresses of everyday life, is able to work productively, and is able to contribute to his or her society” (Saxena and Setoya, 2014). Middle-aged and older adults, especially the elderly, are groups with a high incidence of psychological problems (Finkel et al., 2005; Thompson and Foth, 2005). According to the survey data from National Health Commission of China, in 2019, the mental health rate of the elderly in China was only 28.55% (Miao, 2022). At the same time, the Corona Virus Disease 2019 (COVID-19) pandemic has not only brought an unprecedented impact on peoples’ lives, but also widely affected peoples’ risk perception and exacerbated mental health problems (Zhang, 2022). As a tool that can communicate, obtain, and disseminate information across time and space barriers, as well as a platform for interactive e-commerce, work, study, etc., the Internet has played an important role in response to the COVID-19. According to the 49th “Statistical Report on Internet Development in China” released by China Internet Network Information Center (CNNIC) in 2021, the scale of Chinese netizen has reached 989 million, the Internet penetration rate has reached 70.4%, and the proportion of netizen aged 50 and above reached 26.8% (China Internet Network Information Center, 2022). An increasing number of studies have found that there is a significant correlation between Internet use and psychological health such as loneliness, rejection sensitivity, delay, impulsivity and depression level (Caplan, 2002; Davis et al., 2002; Hossin et al., 2022). Therefore, the penetration of the Internet into the middle-aged and older adults’ groups is bound to have a further impact on their mental health. In addition, some studies argue that the use of the Internet can reduce the general life pressure (LaRose et al., 2001; Shaw and Gant, 2004). Life satisfaction, as an overall assessment of an individual’s feelings and attitudes toward current life, is also one of the important protective factors for perceived stress, which can effectively buffer the impact of adverse factors on mental health (Zheng et al., 2019). However, it is unknown whether life satisfaction is a mediator of the relationship between Internet use and mental health. Under the multiple backgrounds of the in-depth development of information technology, the in-depth popularization of public networks, and active aging becoming a global problem, Internet use and mental health problems of middle-aged and older adults need more scholars’ continuous attention and research.

According to the previous literature, high-level Internet use by older adults can be beneficial with higher levels of mental health (Cotten et al., 2014; Forsman and Nordmyr, 2017; Jun and Kim, 2017). The Internet can be served as a channel to improve the mental health of the elderly (Chen and Persson, 2002; Hernández-Encuentra et al., 2009) by motivating them to obtain mental health treatment (Handley et al., 2015), engage in social activities (Sum et al., 2009), and keep in touch with society (Cangelosi and Sorrell, 2014; Hunsaker et al., 2020; Liao et al., 2020). Additionally, in the virtual world, the elderly can obtain psychological satisfaction and spiritual pleasure, and feel a sense of belonging to the society, which thereby enhances their sense of well-being and improves their mental health (Forsman et al., 2018; Hunsaker and Hargittai, 2018). Previous studies have also found that other factors, such as Internet use ability (Sharit et al., 2008), internet accessibility (Cavender and Bigham, 2011), income level (Chekroud et al., 2018), social class (Ihm and Hsieh, 2015), and family support (Xiong and Zuo, 2019), may affect the mental health of older adults when using the Internet as well.

As the Internet continues to infiltrate daily life, the negative impact of Internet use mental health has attracted extensive attention and research, most of which focus on Internet addiction (Bai et al., 2001; Chou, 2001; Young, 2009) or Internet addiction disorder (IAD; Young et al., 1999). Some researchers argue that excessive use of the Internet may occupy the time and energy originally spent on a healthy lifestyle, leading to lack of exercise and unhealthy eating habits, causing obesity, affecting the normal operation of daily activities, and even causing serious physical and psychological damage (Noel and Epstein, 2003; Kim et al., 2010; Jeong et al., 2020). Some other researchers believe that different Internet use purposes are related to different psychological states. Specifically, people who use the Internet for information have lower life satisfaction (Lam et al., 2020). Internet use for health purposes increases risk of depression, which may be due to unnecessary alarms, or excessive attention to health problems (Bessière et al., 2010). Use of the Internet in study, work, and business has little effect on relieving depression (Yang et al., 2021).

While studying the relationship between Internet use and mental health, some scholars investigated how Internet use affects mental health. They found that internet use enables users to achieve higher levels of mental health by enhancing the following mediators including social capital (Lyu and Sun, 2021; Zhu et al., 2021), frequency of physical activities (Zhang and Zhang, 2021), social integration (Weiser, 2001), and self-efficacy (LaRose et al., 2001; Shaw and Gant, 2004) in using the Internet.

To our knowledge, although there is already a lot of literature about the impact of Internet use on mental health, no consensus has been reached. Most studies only focus on whether Internet use has impact on mental health or not, and do not reflect the impact of specific Internet use purposes and Internet use duration on mental health. Moreover, existing studies have mainly focused on the impact of Internet use on the emotional dimension, and less attention has been paid to the cognitive dimension. Fewer studies have investigated the effect of Internet use on mental health in middle-aged and older adults, using life satisfaction as mediator. Based on the Chinese Family Panel Studies (CFPS) from 2018, the present study aims to (1) investigate the impact of Internet use on the mental health in adults aged 45 years and above; (2) explore the differences in the impact of specific purposes and time of Internet use on the mental health; and (3) determine whether life satisfaction mediate the associations between Internet use and mental health.

## Materials and methods

### Data

The data are derived from the CFPS, a nearly nationwide, comprehensive, longitudinal social survey launched by the ISSS (Institute of Social Science Survey) of Peking University in 2010. The project reflects the changes of Chinese society, economy, population, education and health by tracking and collecting data at three levels: individual, family, and community. CFPS surveyed about 15,000 households from 621 villages/communities in 25 provinces/municipalities/autonomous regions across the country using the “multi-stage probability proportional sampling method,” and interviewed all household members in each sampled household, covering 95% of the population. The CFPS mainly conducted face-to-face interviews aided by computer-assisted personal interviewing (CAPI) technology. In situations where personal interviews were not feasible, computer-assisted telephone interviewing (CATI) technology or computer-assisted Web interviewing (CAWI) technology were used (Xie and Hu, 2015; Xie and Lu, 2015). According to research needs, this paper selects the newer survey data released in 2018.

### Variables

#### Dependent variables

The dependent variable is mental health of middle-aged and older adults. We measure it by using depressed and cognitive function. Depression is a common psychological symptom. Depression level in this study is derived from simplified CES-D scale in CFPS. The abbreviated scale contains eight items from the full CES-D scale: “I am in a low spirit, I find it difficult to do anything, I cannot sleep well, I feel lonely, I feel sad, I feel that I cannot continue with my life, I feel happy, I have a happy life,” and the answers “never (less than 1 day),” “sometimes (1–2 days),” “often (3–4 days),” and “most of the time (5–7 days)” are assigned a value of 1–4 (Among them, the last two items are positive emotions, and the reverse scoring principle is adopted.), the scores of the above questions will be summed up, and the final scores’ value range is 8–32 points. The higher the test score, the higher the depression level. Cognition is the psychological process of obtaining and processing information through sensing, memory, reasoning, and decision-making. Constrained by data availability, literacy-test scores, and math-test scores are used as proxy variables for cognition, and the data came from the cognitive module in the CFPS questionnaire, which administers a literacy-test and a math-test to respondents. The theoretical basis of the test method is the design of the Guttman Scale in psychometrics. In the design of the Guttman Scale, each question can be strictly arranged according to the difficulty of the test questions. According to this order, answering a question correctly means that they can answer all questions that are easier than this question; similarly, answering a question incorrectly means getting all the more difficult questions wrong than this one. In the literacy-test, the interviewer shows respondents 34 Chinese characters from easy to difficult. If the respondents answer three questions incorrectly in a row, the test will be terminated. The sequence position of the most difficult Chinese character that respondents answered correctly determines their final scores. In the math-test, the respondents answered 24 math questions from easy to difficult in sequence. If the respondents answer three questions incorrectly in a row, the test will be terminated. The most difficult objective sequence position that respondents answer correctly determines their scores. In the end, the literacy-test scores range from 0 to 34 points, and the math-test scores range from 0 to 24 points. The higher the score, the stronger the literacy and mathematical calculation skills.

#### Independent variable

The independent variable is Internet use, which refers to the behavior of using mobile phones, computers, and other modern communication technologies for studying, working, socializing, entertainment, shopping, and other activities. This paper mainly measures the Internet use from three dimensions: ① whether to use the Internet; ② Internet use duration; and ③ Internet use purposes. Among them, whether to use the Internet is measured according to the items “Do you use mobile devices, e.g., mobile phone, tablet PC, to access the Internet (Yes/No)” and “Do you use computers to access the Internet (Yes/No)” in CFPS, if the answer is neither use mobile devices nor use a computer to use the Internet, it is assigned the value as 0, otherwise, assigned as 1. The Internet use duration is measured according to the item “In general, how many hours do you spend online in your spare time each week?.” After deleting the missing and inapplicable values, the Internet use duration of the final sample is 0–70 h per week. Internet use purposes includes whether the respondents use the Internet to study, work, socialize, entertain, and conduct business activities. The answers are “never,” “once per a few months,” “once per month,” “2–3 times per month,” “1–2 times per week,” “3–4 times per week,” and “every day,” if the respondent answers “never,” it is assigned as 0, otherwise assigned as 1.

#### Mediating variable

The mediating variable is life satisfaction. It is measured by a question in CFPS: “How would you rate your satisfaction with your life,” with the answer scale of 1–5, and higher scores indicate higher life satisfaction.

#### Covariates

Covariates include the individual’s personal characteristics, socioeconomic status, health status, physical activity, social relationships, and region. Personal characteristics include the individual’s gender, age (45–95 years old), residence, education level (illiterate, primary school, secondary school, and above), and marital status (with and without a spouse). Socioeconomic status includes self-assessed economic status, self-assessed social status, and employment status. The self-assessed economic status and self-assessed social status are scored on a scale of 1–5, with higher scores indicating higher status. The employment status includes three situations: employed, unemployed, and withdrawn from the labor market, among which the employed is assigned a value of 1, and the unemployed and withdrawn from the labor market are assigned a value of 0. Health conditions include chronic diseases and ability to perform daily activities. Chronic disease refers to whether the respondents suffering from chronic disease in the past 6 months, if they do, it is assigned a value of 1, otherwise assigned a value of 0. Activities of daily living are measured by respondents’ independent completion of outdoor activities, meals, kitchen activities, use of public transportation, shopping, cleaning, and laundry, and each activity is scored as 1 point for completion, 0 for failure, and the final scores range from 0 to 7 points, with higher scores indicating better daily activities. Physical activity refers to the number of times respondents exercised in the past week. Social relationships include trust in neighbors, strangers, and personal relationships, each with a score of 0–10, with higher scores indicating higher trust or better personal relationships. According to the division standard of the 2006 China Statistical Yearbook on the three major regions of the eastern, central and western parts of mainland China, the 31 provinces and cities in the mainland are divided into eastern, central, and western regions.

### Statistical analysis

The dependent variables in this paper can be approximately regarded as continuous variables, so we first use multiple linear regressions to estimate the impact of Internet use on mental health, so as to obtain a benchmark result. Then we used Rubin’s counterfactual framework as the theoretical basis, divide the sample into treatment group (netizen) and control group (non-netizen), matched similar samples according to propensity score, and calculated the average processing effect of Internet use on mental health, thereby mitigating the effects of endogeneity (D’Agostino, 1998).

Referring to the classic counterfactual framework, this paper sets a binary dummy variable $D_{i}=\left\{1,0\right\}$, where $D_{i}=1$ means using the Internet, $D_{i}=0$ means not using the Internet, and the mental health status is recorded as $y_{i}$. For individual i, his (or her) mental health status exist the following two states that depends on whether he (or her) uses the Internet or not:


(1) $$y_{i}\{\begin{array}{l} y_{1i}\;\mathrm{if}\;D_{i}=1\\ y_{0i}\;\mathrm{if}\;D_{i}=0 \end{array}$$

In formula (1), $y_{1i}$ represents the mental health status of using the Internet, $y_{0i}$represents the mental health status of not using the Internet, and the following formula can be obtained by further converting it into a piece-wise function:


(2) $$y_{i}=\left(1-D_{i}\right)y_{0i}+D_{i}y_{1i}=y_{0i}+\left(y_{1i}-y_{0i}\right)D_{i}$$

In formula (2), $\left(y_{1i}-y_{0i}\right)$ represents the processing effect of individual i use of the Internet.

This paper focuses on the processing effect of using the Internet on the mental health of middle-aged and older adults, so the average processing effect of the treatment group is selected for analysis, and the expression is as following:


(3) $$ATT=E\left(y_{1i}-y_{0i}|D_{i}=1\right)=E\left(y_{1i}|D_{i}=1\right)-E\left(y_{0i}|D_{i}=1\right)$$

In formula (3), $y_{1i}$ represents the depression scores or literacy-test scores and math-test scores of the individual using the Internet, $y_{0i}$ represents the depression scores or literacy-test scores and math-test scores if the individual does not use the Internet,$y_{1i}-y_{0i}$ represents the processing effect of the individual using the Internet, and ATTrepresents the difference between the depression scores (or literacy-test scores and math-test scores) of middle-aged and older adults who use the Internet and the depression (or literacy-test scores and math-test scores) if they do not use the Internet. Among them, $E\left(y_{0i}|D_{i}=1\right)$ is a counterfactual result, which cannot be observed in reality, and the basic idea of the propensity score matching method is to find a $E\left(y_{0i}|D_{i}=1\right)$ that can replace $E\left(y_{0i}|D_{i}=0\right)$ to complete the counterfactual estimation.

Secondly, we further analyze the impact of Internet use duration and different Internet purposes on mental health. When analyzing the impact of Internet use duration on mental health, we include Internet use duration and its quadratic term in the linear model for regression, and combine the U-test method to test the U-shaped relationship between Internet use duration and mental health.

Finally, we select the stepwise test regression coefficient method and the bootstrap method to further verify the mediating role of life satisfaction in the process of Internet use affecting mental health (Wen and Ye, 2014). The mediation test model is set as follows:


(4) $$Y_{i}=cI_{i}+\varepsilon_{1}$$

(5) $$M_{i}=aI_{i}+\varepsilon_{2}$$

(6) $$Y_{i}=c^{\prime }I_{i}+bM_{i}+\varepsilon_{3}$$

In formula (4), (5), and (6), i is the same as described above. $Y_{i}$ represents the dependent variables. $I_{i}$ represents the independent variables.$M_{i}$ is the mediating variable life satisfaction, c is the total effect of using the Internet on depression or literacy-test scores and math-test scores, $c^{\prime }$ is the direct effect, a is the relationship between Internet use and life satisfaction, ab is the mediating effect, $\varepsilon_{1}$, $\varepsilon_{2}$, and $\varepsilon_{3}$ are the error terms.

## Results

### Sample description

Table 1 reports the descriptive statistics of the main variables and differences in Internet usage in this study. As can be seen from the table, this study obtained a total of 14,497 middle-aged and older adults’ samples, including 3,906 samples from netizen and 10,591 samples from non-netizen. Netizen spend an average of 10.60 h a week on the Internet in their spare time. The proportion of netizen who use the Internet to socialize interaction and entertain is the highest, followed by use the Internet to study and business activities, and the lowest proportion of work. The mean scores of depression scores and life satisfaction in the netizen group are lower than those in the non-netizen group, while the scores on the literacy-test and math-test are higher than those in the non-netizen group. In terms of gender distribution, the proportion of males in the netizen group is higher than that of the non-netizen group. In terms of age, the average age of the netizen group is significantly lower than that of the non-netizen group. In terms of place of residence, most of the netizen group are urban population, while most of the non-netizen group are rural population. In terms of education level, the proportion of illiterate people and primary school education in the netizen group is lower than that of the non-netizen group, but the proportion of middle school and above is significantly higher than that of the non-netizen group. In terms of marital status, the netizen group has a higher proportion of spouses. In terms of socioeconomic status, the self-assessed economic status and self-assessed social status of the netizen group are significantly lower than those of the non-netizen group. In terms of employment status, the proportion of netizen who have jobs is higher. In terms of health status, the netizen group has a higher prevalence of chronic diseases, but also has a higher average score on daily activities. In terms of exercise situation, the average frequency of physical exercise in the netizen group is higher. In terms of social relations, the netizen group has higher trust in strangers, but lower trust in neighbors and poorer personal relationships. From the perspective of regional distribution, both the netizen group and the non-netizen group are the majority of the eastern population.

**Table 1:** Sample description.

**Variable**	**Full sample (*N* = 14,497)**	**Netizen (*N* = 3,906)**	**Non-netizen (*N* = 10,591)**
Internet use duration, mean (SD)	2.86 (7.00)	10.60 (9.99)	–
Studying, %	9.74	36.15	–
Working, %	6.90	25.60	–
Social interaction, %	22.48	83.44	–
Entertainment, %	21.64	80.31	–
Business activities, %	10.34	38.38	–
Depression scores, mean (SD)	13.78 (4.32)	13.12 (3.82)	14.03 (4.47)
Literacy-test scores, mean (SD)	15.44 (10.37)	22.64 (7.68)	12.79 (9.97)
Math-test scores, mean (SD)	6.46 (4.39)	9.41 (4.29)	5.37 (3.90)
Life satisfaction, mean (SD)	4.13 (0.96)	3.99 (0.92)	4.18 (0.98)
Gender, %	49.31	53.84	47.63
Age, mean (SD)	59.53 (9.75)	53.95 (7.56)	61.58 (9.67)
Town, %	46.84	63.06	40.89
Illiteracy, %	35.29	10.93	44.27
Primary school, %	25.79	21.97	27.20
Secondary school and above, %	38.92	67.10	28.52
Married, %	87.73	91.99	86.16
Self-assessed economic status, mean (SD)	2.98 (1.14)	2.87 (1.00)	3.02 (1.18)
Self-assessed social status, mean (SD)	3.29 (1.12)	3.09 (1.01)	3.36 (1.15)
Employed, %	69.60	73.27	68.25
Chronic disease, %	75.20	78.57	73.96
Daily activities, mean (SD)	3.06 (3.55)	3.57 (3.28)	2.88 (3.63)
Physical activity, mean (SD)	6.68 (1.00)	6.91 (0.52)	6.60 (1.12)
Trust in neighbors, mean (SD)	6.82 (2.18)	6.75 (2.01)	6.85 (2.24)
Trust in strangers, mean (SD)	2.03 (2.23)	2.13 (2.10)	1.99 (2.27)
Relationship, mean (SD)	7.24 (2.07)	7.17 (1.83)	7.26 (2.15)
Eastern Region, %	42.78	46.19	41.53
Central Region, %	28.65	28.80	28.59
Western Region, %	28.57	25.01	29.88

### Relationship between Internet use and mental health

The depression scores, literacy-test, and math-test scores are included in the multivariate linear model for regression analysis, respectively. As shown in Table 2, the goodness of fit of each model is very good (*R*^2^ were, respectively, 0.17, 0.56, and 0.48), and the joint significance of the regression coefficient is also very high (Prob>*F* = 0.00). This suggests that individual depression scores, literacy-test and math-test scores are all well predicted by Internet use, controlling for other variables. From the results in Table 2, Internet use has a significant impact on depression scores, literacy-test scores, and math-test scores: middle-aged and older adults who use the Internet have lower depression scores, while literacy-test scores and math-test scores are higher. And through comparison, we can find that the impact of Internet use on the scores of the literacy-test (coefficient = 3.88) and the scores of the math-test (coefficient = 1.63) is much greater than that of depression scores (coefficient = −0.26).

**Table 2:** Relationship between Internet use and mental health.

**Variable**	**Depression scores**	**Literacy-test scores**	**Math-test scores**
Internet use	−0.26^***^	3.88^***^	1.63^***^
Gender	−1.00^***^	2.13^***^	0.88^***^
Age	−0.02^***^	−0.06^***^	−0.03^***^
Town	−0.73^***^	1.44^***^	0.47^***^
Primary school	−0.52^***^	9.48^***^	3.05^***^
Secondary school and above	−0.87^***^	13.34^***^	5.25^***^
Married	−1.54^***^	0.06	0.17^**^
Self-assessed economic status	−0.30^***^	−0.16^***^	−0.09^***^
Self-assessed social status	−0.19^***^	−0.28^***^	−0.15^***^
Employed	0.17^**^	−0.70^***^	−0.07
Chronic disease	−0.36^***^	−0.13^***^	−0.05^***^
Daily activities	−0.58^***^	0.68^***^	0.28^***^
Physical activity	−0.06^***^	0.08^***^	0.03^***^
Trust in neighbors	−0.19^***^	0.03	0.02^*^
Trust in strangers	0.03^*^	−0.01	0.05^***^
Relationship	−0.16^***^	−0.01	0.01
Eastern region	−0.92^***^	1.69^***^	0.56^***^
Western region	−0.66^***^	1.15^***^	0.36^***^
constant	27.72^***^	5.09^***^	2.23^***^
*R* ^2^	0.17	0.56	0.48
Prob>F	0.00	0.00	0.00
*N*	14,497	14,497	14,497

The numbers in the table represent the influence coefficient; ^***^p < 0.01, ^**^p < 0.05, and ^*^p < 0.1.

In addition to Internet use, some covariates also significantly affect the mental health of middle-aged and older adults. Men score lower on depression and higher on literacy-test and math-test than women. Older age is associated with lower depression scores, as well as lower literacy-test and math-test scores. Urban middle-aged and older adults have lower depression scores than rural middle-aged and older adults, and higher scores on literacy-test and math-test. Middle-aged and older adults with a primary and secondary education or higher had lower depression scores than illiterate middle-aged and older adults, and higher scores on literacy-test and math-test. Middle-aged and older adults with spouses score lower on depression and higher on literacy-test and math-test. Middle-aged and older adults with higher self-assessed economic and social status have lower depression scores, but also lower scores on literacy-test and math-test. Middle-aged and older adults with jobs score higher on depression and on literacy-test. Middle-aged and older adults with chronic illnesses score lower on depression, as well as on literacy-test and math-test. Middle-aged and older adults with better daily activities had lower depression scores and higher scores on literacy-test and math-test. Physical activity frequency has a significant negative effect on depression scores and a positive effect on literacy-test and math-test scores. Middle-aged and older adults with higher frequency of physical activity have lower depression scores and higher scores on literacy-test and math-test. Middle-aged and older adults with higher trust in their neighbors have lower depression scores and higher math-test scores. Middle-aged and older adults with higher trust in strangers have higher depression scores and higher math-test scores. Relationships have a significant negative effect on depression scores, but have no significant effect on literacy-test and math-test scores. The better the relationship, the lower the depression scores. Middle-aged and older adults in the East and West score lower on depression and higher on literacy-test and math-test.

### PSM analysis of Internet use and mental health

**Figure 1:**
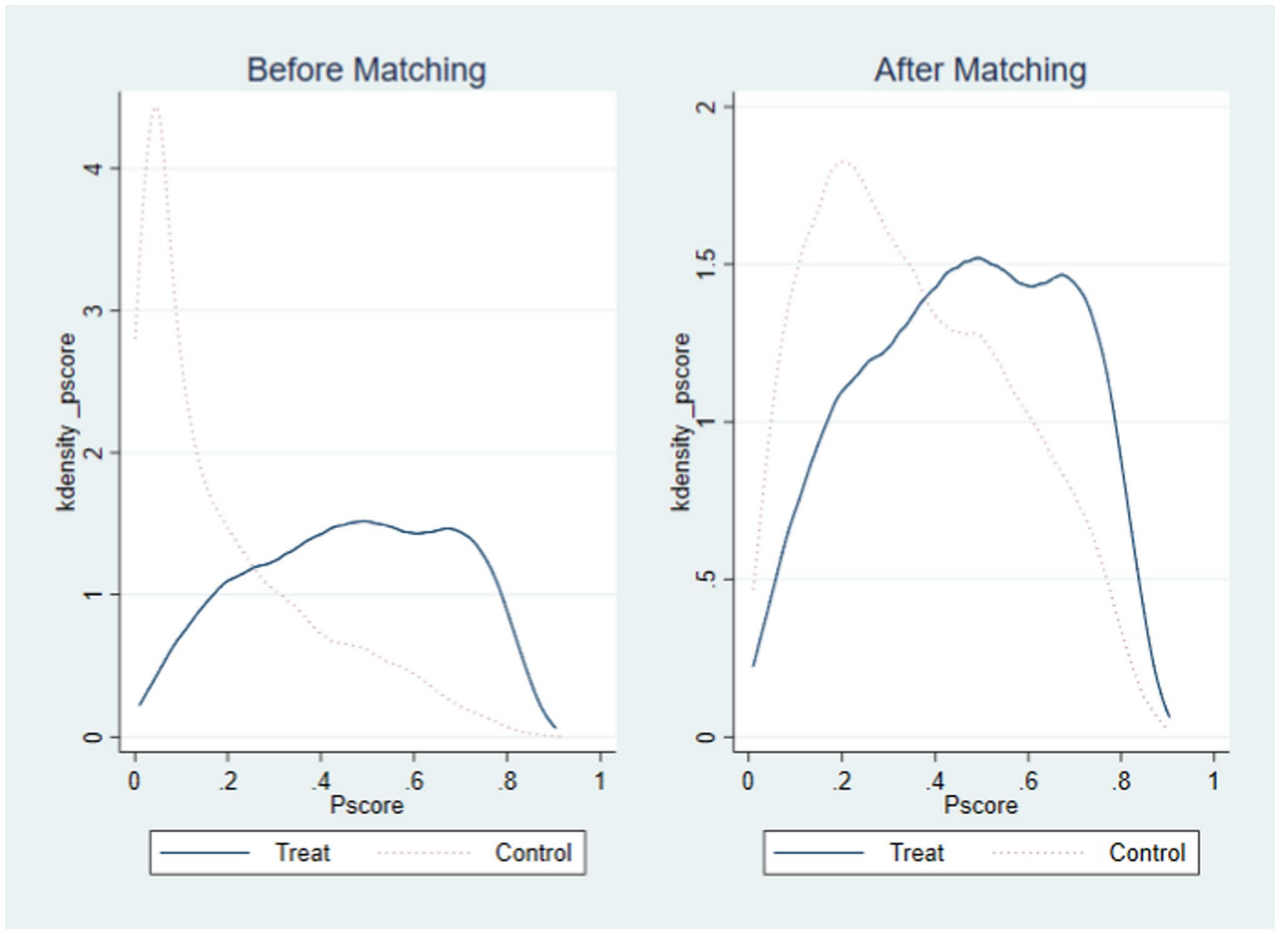
Kernel density distribution before and after matching.

**Figure 2:**
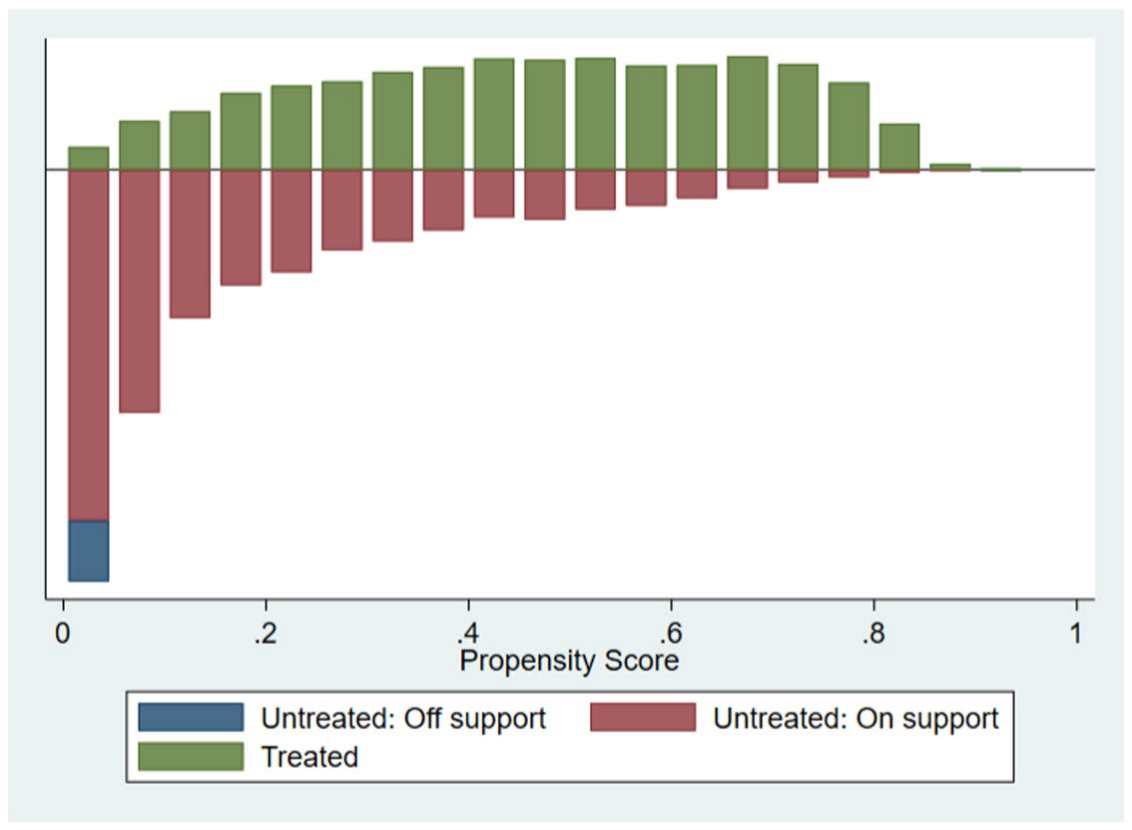
Common support hypothesis test.

The first step of the propensity score matching is to obtain the key basis for matching, that is, the propensity score of respondents for choosing to use the Internet. This paper uses the logit model to estimate the propensity scores of respondents to use the Internet. Next, the matching effect test and the common support test are carried out. The sample matching results show that after matching, the absolute value of the standardized deviation of the variables is less than 10% (Rosenbaum and Rubin, 1985), The *t*-values are significant before matching, but not significant after matching. The above results show that there is no systematic difference between the post-matching treatment group and the control group, which effectively solves the endogeneity bias caused by the sample selection bias. As shown in the comparison chart of nuclear density distribution in Figure 1, before matching, the nuclear density equation curves of the treatment group and the control group are quite different. After matching, the difference between the nuclear density equation curves of the treatment group and the control group decrease, and the trends tend to be consistent. It can also be seen from Figure 2 that most of the samples are successfully matched, and they are all matched within the common value range, which indicate that the matching quality is good. Since the results of radius matching, kernel matching, and k-nearest neighbor matching methods are not much different. Due to space limitations, only the results of k-nearest neighbor matching are given here. Finally, the processing effects of Internet use in middle-aged and older adults on their depression scores, literacy-test scores, and math-test scores are calculated.

Table 3 reports the results of treatment effect calculated by propensity score matching. The results show that after controlling for endogenous bias, the effect of Internet use on depression scores, literacy-test scores and math-test scores is still very significant. The computation results using the three methods of *k*-nearest neighbor matching, radius matching, and kernel matching show that the processing effect of using the Internet on depression scores is from −0.29 to −0.33, and the processing effects on literacy-test scores and math-test scores are from 3.60 to 3.68 and from 1.72 to 1.73 respectively, which proves that the use of the Internet can reduce depression level to a certain extent, and improve the ability of literacy and mathematical computation.

**Table 3:** PSM analysis of Internet use and mental health.

**Matching method**	**Sample**	**Depression scores**	**Literacy-test scores**	**Math-test scores**
K-nearest neighbor	Unmatched	−0.91 (0.08)^***^	9.85 (0.18)^***^	4.04 (0.08)^***^
	Matched	−0.33 (0.11)^***^	3.68 (0.23)^***^	1.72 (0.11)^***^
Radius matching	Unmatched	−0.91 (0.08)^***^	9.85 (0.18)^***^	4.04 (0.08)^***^
	Matched	−0.29 (0.10)^***^	3.60 (0.22)^***^	1.72 (0.10)^***^
Kernel matching	Unmatched	−0.91 (0.08)^***^	9.85 (0.18)^***^	4.04 (0.08)^***^
	Matched	−0.31 (0.10)^***^	3.67 (0.22)^***^	1.73 (0.10)^***^

Values outside brackets represent ATT, and values in brackets represent standard deviation. ^***^p < 0.01.

### Further analysis

#### Relationship between Internet use duration and mental health

From the results reported in Table 4, when depression score is the dependent variable, after including the quadratic term of Internet use duration, the coefficient of the first term of Internet use duration is still negative and significant, while the coefficient of the quadratic term is positive and significant. When literacy-test and math-test scores are used as dependent variables, after including the quadratic term of Internet use duration, the coefficient of the first term of Internet use duration is still positive and significant, while the coefficient of the quadratic term is negative and significant. The above results show that there is a U-shaped relationship between Internet use duration and depression, and an inverted U-shaped relationship between higher scores in literacy-test and math-test. However, it is too weak to judge whether it is a U-shaped only by the positive or negative of the quadratic coefficient. When the real relationship is convex and monotonic, the model estimation will erroneously generate an extreme point and a U-shaped relationship (Lind and Mehlum, 2010). Therefore, we continued to use the U-test invented by Sasabuschi, Lind and Mehlum to verify the relationship between Internet use duration and depression scores, literacy-test scores and math-test scores (Sasabuchi, 1980; Haans et al., 2016).

**Table 4:** Relationship between Internet use duration and mental health.

	**Depression scores**		**Literacy-test scores**		**Math-test scores**	
Internet use duration	−0.02^**^	−0.04^***^	0.18^***^	0 0.37^***^	0.08^***^	0.17^***^
Internet use duration^2		0.001^**^		−0.006^***^		−0.003^***^
Control variables	YES	YES	YES	YES	YES	YES
*N*	14,497	14,497	14,497	14,497	14,497	14,497
Prob > F	0.00	0.00	0.00	0.00	0.00	0.00
*R* ^2^	0.17	0.17	0.55	0.55	0.48	0.48

Numbers marked with ^*^represent influence coefficients, ^***^p < 0.01, ^**^p < 0.05.

As shown in Table 5, the U-test test results of Internet use duration and depression score show that the upper and lower bounds of Slope are positive and negative, respectively, and the extreme point is 26.34, which is between the upper and lower bounds. The value of *p* for overall test of presence of a U shape is 0.004, which significantly reject the null hypothesis, so there is a U-shaped relationship between the duration of Internet use and depression scores. The U-test results of Internet use duration and literacy-test scores show that Slope is positive and negative at the upper and lower bounds, respectively, and the extreme point is 32.85, which is between the upper and lower bounds. The value of *p* for overall test of presence of a U-shape is 6.52e-18, which significantly reject the null hypothesis, so there is an inverted U-shaped relationship between the Internet use duration and the literacy-test scores. The U-test results of Internet use duration and math-test scores show that Slope is positive and negative at the upper and lower bounds, respectively, and the extreme point is 31.25, which is also between the upper and lower bounds. The value of *p* for overall test of presence of a U-shape is 1.02e-20, which significantly rejected the null hypothesis, so there was an inverted U-shaped relationship between Internet use duration and the math-test scores.

**Table 5:** U-test.

	**Depression scores**		**Literacy-test scores**		**Math-test scores**	
	Lower bound	Upper bound	Lower bound	Upper bound	Lower bound	Upper bound
*t* value	−4.43	2.63	21.10	−8.55	20.87	−9.27
Interval	0	70	0	70	0	70
Slope	−0.02	0.04	0.37	−0.42	0.17	−0.21
*P* > |*t*|	0.004		6.52e−18		1.02e−20	
Extreme point	26.34		32.85		31.25	

#### Relationship between Internet use purposes and mental health

From the estimated results in Table 6, the results of the three matching methods all show that use the Internet for entertaining and business activities has a significant impact on depression scores. Middle-aged and older adults who use the Internet for entertaining and business activities have lower depression scores than those who do not use the Internet for entertaining or business activities. However, the use of the Internet for studying, working, and social interaction has no significant effect on the depression scores. In contrast, use of the Internet for studying, working, social interaction, entertaining, and business activities is significantly associated with both literacy-test and math-test scores at the 1% level. Middle-aged and older adults who use the Internet for studying, working, social interaction, entertaining, and business activities have higher scores on literacy-test and math-test.

**Table 6:** Relationship between Internet use purposes and mental health.

Internet use purposes	Matching method	Depression scores	Literacy-test scores	Math-test scores
Studying	K-nearest neighbor	−0.14 (0.13)	3.96 (0.23)^***^	2.43 (0.14)^***^
	Radius matching	−0.13 (0.12)	4.15 (0.22)^***^	2.42 (0.13)^***^
	Kernel matching	−0.15 (0.11)	4.48 (0.22)^***^	2.56 (0.13)^***^
Working	K-nearest neighbor	0.01 (0.15)	3.65 (0.29)^***^	2.23 (0.18)^***^
	Radius matching	−0.01 (0.15)	3.67 (0.30)^***^	2.25 (0.17)^***^
	Kernel matching	0.07 (0.14)	4.03 (0.29)^***^	2.39 (0.17)^***^
Social interaction	K-nearest neighbor	−0.18 (0.11)^*^	3.31 (0.23)^***^	1.54 (0.11)^***^
	Radius matching	−0.23 (0.10)^**^	3.33 (0.22)^***^	1.60 (0.10)^***^
	Kernel matching	−0.25 (0.10)^**^	3.45 (0.21)^***^	1.64 (0.10)^***^
Entertainment	K-nearest neighbor	−0.26 (0.11)^**^	3.39 (0.23)^***^	1.50 (0.11)^***^
	Radius matching	−0.29 (0.10)^***^	3.44 (0.22)^***^	1.54 (0.10)^***^
	Kernel matching	−0.28 (0.10)^***^	3.52 (0.21)^***^	1.58 (0.10)^***^
Business activities	K-nearest neighbor	−0.27 (0.13)^**^	3.18 (0.26)^***^	1.82 (0.15)^***^
	Radius matching	−0.25 (0.12)^**^	3.21 (0.25)^***^	1.88 (0.14)^***^
	Kernel matching	−0.29 (0.12)^**^	3.49 (0.25)^***^	1.99 (0.13)^***^

Values outside brackets represent ATT, and values in brackets represent standard deviation. ^***^p < 0.01, ^**^p < 0.05, ^*^p < 0.1.

### Mediating effect of life satisfaction

Table 7 reports the results of analyzing the mediation effect using a two-step regression method (Zhao et al., 2010). The first step is to analyze the relationship between Internet use and life satisfaction. The results show that the use of the Internet significantly reduces the life satisfaction of middle-aged and older adults (the coefficient was −0.04). The second step is to add depression score, literacy-test score and math-test score as explained variables into regression model to analyze the relationship between independent variables, mediator variables and dependent variables. The results show that the coefficients of Internet use and life satisfaction are very significant, and the indirect effect values obtained by calculating the multiplication of coefficients are all positive numbers (respectively: 0.01, 0.01, and 0.01), indicating that the mediating effect is significant. However, the direct effect (−0.91) of Internet use on depression scores has the opposite sign to the indirect effect, that is, the nature of the mediating effect here is “masking effect” (MacKinnon et al., 2000), while the direct effect on literacy-test and math-test scores has the same sign as the indirect effect, that is, there are some intermediaries. To test the robustness of the above results, we use the bias-corrected nonparametric percentile bootstrap method to calculate the 95% confidence interval (Bias-corrected 95% CI) to further verify the mediation effect. As shown in Table 8, the 95% confidence interval after bias correction does not include 0, and the coefficient product is significant, so there is a mediating effect (Preacher et al., 2007; Preacher and Hayes, 2008). That is, life satisfaction has a masking effect between Internet use and depression scores, and there is a partial mediation between Internet use and literacy-test scores and math-test scores. This is consistent with the results obtained by the stepwise test coefficient method.

**Table 7:** Mediating effect of life satisfaction.

	Life satisfaction	Depression scores	Literacy-test scores	Math-test scores
Internet use	−0.04^**^	−0.91^***^	0.97^***^	0.77^***^
Life satisfaction		−0.29^***^	−0.30^***^	−0.32^***^
Control variables	YES	YES	YES	YES
*N*	14,525	14,289	14,525	14,525
*R* ^2^	0.12	0.13	0.25	0.28

Numbers marked with ^*^represent influence coefficients, ^***^p < 0.01, ^**^p < 0.05.

**Table 8:** Mediating effect of life satisfaction.

	**Depression scores**	**Literacy-test scores**	**Math-test scores**
Indirect effect	0.03 (0.001 ~ 0.07)	0.006 (0.001 ~ 0.02)	0.004 (0.001 ~ 0.01)
Direct effect	−0.29 (0.44 ~ −0.14)	3.88 (3.61 ~ 4.19)	1.63 (1.49 ~ 1.75)

The figures in the table are the mediation effect coefficient and bias-corrected 95% CI.

## Discussion

Based on the 2018 CFPS survey data, this study comprehensively used multiple linear regression, PSM, stepwise test coefficient method and bootstrap method to explore the impact of Internet use on the mental health of middle-aged and older adults in China and its mediating effect. The results found that, compared with never using the Internet, moderate use of the Internet can significantly reduce the level of depression in middle-aged and older adults, promote their cognitive function, and improve their mental health, which is consistent with previous research findings. Using the Internet can enable middle-aged and older adults to obtain health knowledge and network services (Cohall et al., 2011; Zhao and Liu, 2020), relieve their loneliness (van Boekel et al., 2017; Zhaoran et al., 2017) and depression level, thereby improving their mental health. But excessive internet use will negatively impact the mental health. Previous studies also believe that with the increase of Internet use duration, the user’s social participation and mental health level gradually decreased, and the level of loneliness, depression and psychological cognitive burden gradually increased (Kraut et al., 1998; Jung et al., 2016). Excessive use of the Internet can also effectively predict individual psychosocial health such as loneliness, rejection sensitivity, delay, and impulsivity (Caplan, 2002; Davis et al., 2002; Hossin et al., 2022). In-depth analysis also found that online socializing, entertainment and business activities have a significant alleviating effect on the level of depression, but online study and work did not have a significant impact on the level of depression. Because playing online games, watching short videos, listening to music and other online entertainment activities and online business activities such as shopping can bring direct and high-frequency mental stimulation to middle-aged and older adults, as well as instant audio-visual satisfaction, so that they can quickly vent their emotions and release pressure (Liu et al., 2013; Jung and Kim, 2016). Online study, work, social interaction, entertainment and business activities all contribute to the cognitive function. Because these processes usually require users to continuously process information and maintain thinking activities, which will have an effect on their thinking, memory, attention and emotional regulation (Wilmer et al., 2017). Overall, the above results indicate that different purposes of Internet use and time spent online are related to different psychological states. When considering the impact of Internet use on mental health, it is necessary to assess how differences in different purposes of Internet use and time spent online moderate this effect.

In addition, we used life satisfaction as a mediating variable, and used the stepwise test coefficient method and bootstrap test to analyze its mediating effect on the relationship between Internet use and depression level, literacy ability, and mathematical calculation ability. Unexpectedly, life satisfaction partially suppressed the effect of Internet use on depression and partially mediated the effect of Internet use on literacy and mathematical numeracy. The reason for the above effect may be that (1) Internet use blurs the class cognition of middle-aged and older adults’ groups through reference objects and online language, which makes them feel relatively deprived in social comparison, reduces their subjective class identity (Senik, 2011; Feng and Liu, 2022), and affects their lives (Austin et al., 1977); (2) The “risk amplification effect” and “replacement trauma effect” of the Internet will affect users’ social risk perception (Xu and Lai, 2021), resulting in negative social psychological effects to a certain extent (Schiffrin, 2015; Gimpelson and Treisman, 2018); (3) Internet use may also reduce the social scale of middle-aged and older adults (Senik, 2011) and reduce social trust (Sabatini and Sarracino, 2017), resulting in low life satisfaction. And lower life satisfaction tends to imply greater perceived stress, which predisposes to higher depression levels and predisposes respondents to demonstrate high levels of concentration and concentration on literacy-test and math-test (Strauss and Allen, 2009; Schücker et al., 2013), resulting in higher scores.

## Conclusion and limitation

In general, moderate use of the Internet can significantly relieve depression in middle-aged and older adults and help them maintain cognitive function. However, excessive use of the Internet will also lead to an increase in depression levels and a decline in cognitive function, and different purposes of the Internet will lead to different results. In addition, Internet use reduces life satisfaction, and thus has an impact on mental health.

The contribution of this study is to explore the influence of Internet use and its purpose and duration on different dimensions of mental health, enriching the related research on Internet use and mental health, and has important theoretical significance. In addition, the trend of China’s aging society is becoming more and more serious, and the current digital divide has become a new problem that hinders the elderly from integrating into the new digital life and enjoying their old age. Based on the concept and intention of active aging, this study explores the impact of Internet use on the mental health of the elderly, which will help to provide empirical reference for the formulation of relevant policies, so as to better use network resources to improve the mental health of middle-aged and older adults. Therefore, this study also has important practical significance. The limitations of this study are: on the one hand, in order to meet the overall research needs, the data used in this paper are cross-sectional data in 2018, the Internet usage and mental health status of middle-aged and older adults may be slightly different from the current ones. It is also impossible to estimate trends in mental health with changes in Internet usage from cross-sectional data. On the other hand, the purpose of using the Internet in this article only involves studying, working, socializing, entertaining and conducting business activities, and may omit some other purposes of using the Internet. Therefore, it is expected that the following scholars can draw different results and analyses based on updated longitudinal survey data and more complete variable measurement.

## Data availability statement

Publicly available datasets were analyzed in this study. This data can be found at: the datasets presented in this study can be found in online repositories. The names of the repository/repositories and accession number(s) can be found at: https://opendata.pku.edu.cn/dataverse/CFPS?language=en.

## Ethics statement

The studies involving human participants were reviewed and approved by Peking University Biomedical Ethics Committee. Written informed consent to participate in this study was provided by the participants’ legal guardian/next of kin. The patients/participants provided their written informed consent to participate in this study. Written informed consent was obtained from the individual(s) for the publication of any potentially identifiable images or data included in this article.

## Author contributions

CZ is responsible for the empirical analysis, full text drafting, conclusion, and article revision. YW is responsible for the data, variables, and methods. JW is responsible for the literature review. XL is responsible for the introduction. All authors contributed to the article and approved the submitted version.

## Funding

This work was supported by the University of Electronic Science and Technology of China Scientific Research Start-up Fund (Grant No. Y030222059002015). The funding body has no role in the design of the study, data collection, analysis, interpretation of the data, and write up of the manuscript.

## Conflict of interest

The authors declare that the research was conducted in the absence of any commercial or financial relationships that could be construed as a potential conflict of interest.

## Publisher’s note

All claims expressed in this article are solely those of the authors and do not necessarily represent those of their affiliated organizations, or those of the publisher, the editors and the reviewers. Any product that may be evaluated in this article, or claim that may be made by its manufacturer, is not guaranteed or endorsed by the publisher.

## References

[ref1] Austin G. Preston D. Stewart W. Baldwin E. Riggins G. Salyer K. M. (1977). Some perspectives on compensatory education and inequality. Contemp. Educ. Psychol. 2, 311–320. doi: 10.1016/0361-476X(77)90034-0

[ref2] Bai Y. M. Lin C. C. Chen J. Y. (2001). Internet addiction disorder among clients of a virtual clinic. Psychiatr. Serv. 52: 1397. doi: 10.1176/appi.ps.52.10.1397 11585966

[ref3] Bessière K. Pressman S. Kiesler S. Kraut R. (2010). Effects of internet use on health and depression: a longitudinal study. J. Med. Internet Res. 12:e1149. doi: 10.2196/jmir.1149 PMC3234167 20228047

[ref4] Cangelosi P. R. Sorrell J. M. (2014). Use of technology to enhance mental health for older adults. J. Psychosoc. Nurs. Ment. Health Serv. 52, 17–20. doi: 10.3928/02793695-20140721-01 25062353

[ref5] Caplan S. E. (2002). Problematic internet use and psychosocial well-being: development of a theory-based cognitive–behavioral measurement instrument. Comput. Hum. Behav. 18, 553–575. doi: 10.1016/S0747-5632(02)00004-3

[ref6] Cavender A. C. Bigham J. P. (2011). Toward web accessibility for older users. Univ. Access Inf. Soc. 10, 357–358. doi: 10.1007/s10209-011-0219-y

[ref7] Chekroud S. R. Gueorguieva R. Zheutlin A. B. Paulus M. Krumholz H. M. Krystal J. H.. (2018). Association between physical exercise and mental health in 1· 2 million individuals in the USA between 2011 and 2015: a cross-sectional study. Lancet Psychiatry 5, 739–746. doi: 10.1016/S2215-0366(18)30227-X 30099000

[ref8] Chen Y. Persson A. (2002). Internet use among young and older adults: relation to psychological well-being. Educ. Gerontol. 28, 731–744. doi: 10.1080/03601270290099921

[ref9] China Internet Network Information Center (2022). The 49th Statistical Report on China’ Internet Development. Available at: http://www.cnnic.net.cn/hlwfzyj/hlwxzbg/hlwtjbg/202202/t20220225_71727.htm (Accessed July 20, 2022).

[ref10] Chou C. (2001). Internet heavy use and addiction among Taiwanese college students: an online interview study. CyberPsychol. Behav. 4, 573–585. doi: 10.1089/109493101753235160 11725650

[ref11] Cohall A. T. Nye A. Moon-Howard J. Kukafka R. Dye B. Vaughan R. D.. (2011). Computer use, internet access, and online health searching among Harlem adults. Am. J. Health Promot. 25, 325–333. doi: 10.4278/ajhp.090325-QUAN-121 21534835

[ref12] Cotten S. R. Ford G. Ford S. Hale T. M. (2014). Internet use and depression among retired older adults in the United States: a longitudinal analysis. J. Gerontol. B Psychol. Sci. Soc. Sci. 69, 763–771. doi: 10.1093/geronb/gbu018 24671896

[ref13] D’Agostino R. B. (1998). Propensity score methods for bias reduction in the comparison of a treatment to a non-randomized control group. Stat. Med. 17, 2265–2281. doi: 10.1002/(SICI)1097-0258(19981015)17:19<2265::AID-SIM918>3.0.CO;2-B 9802183

[ref14] Davis R. A. Flett G. L. Besser A. (2002). Validation of a new scale for measuring problematic internet use: implications for pre-employment screening. CyberPsychol. Behav. 5, 331–345. doi: 10.1089/109493102760275581 12216698

[ref15] Feng T. Y. Liu Y. (2022). The lmpact of internet use on the subjective class identity of the elderly in the digital age. J. Xi'an Jiaotong Univ. 42, 122–131. doi: 10.15896/j.xjtuskxb.202202013

[ref16] Finkel D. Reynolds C. A. McArdle J. J. Pedersen N. L. (2005). The longitudinal relationship between processing speed and cognitive ability: genetic and environmental influences. Behav. Genet. 35, 535–549. doi: 10.1007/s10519-005-3281-5 16184483

[ref17] Forsman A. K. Nordmyr J. (2017). Psychosocial links between internet use and mental health in later life: a systematic review of quantitative and qualitative evidence. J. Appl. Gerontol. 36, 1471–1518. doi: 10.1177/0733464815595509 26245208

[ref18] Forsman A. K. Nordmyr J. Matosevic T. Park A. L. Wahlbeck K. McDaid D. (2018). Promoting mental wellbeing among older people: technology-based interventions. Health Promot. Int. 33, 1042–1054. doi: 10.1093/geroni/igx004.4318 28973587

[ref19] Gimpelson V. Treisman D. (2018). Misperceiving inequality. Econ. Polit. 30, 27–54. doi: 10.1111/ecpo.12103

[ref20] Haans R. F. Pieters C. He Z. L. (2016). Thinking about U: theorizing and testing U-and inverted U-shaped relationships in strategy research. Strateg. Manag. J. 37, 1177–1195. doi: 10.1002/smj.2399

[ref21] Handley T. Perkins D. Kay-Lambkin F. Lewin T. Kelly B. (2015). Familiarity with and intentions to use internet-delivered mental health treatments among older rural adults. Aging Ment. Health 19, 989–996. doi: 10.1080/13607863.2014.981744 25420968

[ref22] Hernández-Encuentra E. Pousada M. Gómez-Zúñiga B. (2009). ICT and older people: beyond usability. Educ. Gerontol. 35, 226–245. doi: 10.1080/03601270802466934

[ref23] Hossin M. Z. Islam A. Billah M. Haque M. Uddin J. (2022). Is there a gradient in the association between internet addiction and health? PLoS One 17:e0264716. doi: 10.1371/journal.pone.0264716 35239733 PMC8893621

[ref24] Hunsaker A. Hargittai E. (2018). A review of internet use among older adults. New Media Soc. 20, 3937–3954. doi: 10.1177/1461444818787348

[ref25] Hunsaker A. Hargittai E. Piper A. (2020). Online social connectedness and anxiety among older adults. Int. J. Commun. 14, 697–725.

[ref26] Ihm J. Hsieh Y. P. (2015). The implications of information and communication technology use for the social well-being of older adults. Inf. Commun. Soc. 18, 1123–1138. doi: 10.1080/1369118X.2015.1019912

[ref27] Jeong Y. W. Han Y. R. Kim S. K. Jeong H. S. (2020). The frequency of impairments in everyday activities due to the overuse of the internet, gaming, or smartphone, and its relationship to health-related quality of life in Korea. BMC Public Health 20, 1–16. doi: 10.1186/s12889-020-08922-z 32552690 PMC7301989

[ref28] Jun H. J. Kim M. Y. (2017). What accounts for the relationship between internet use and suicidal ideation of Korean older adults? A mediation analysis. J. Gerontol. B Psychol. Sci. Soc. Sci. 72, 846–855. doi: 10.1093/geronb/gbw163 28025280

[ref29] Jung M. S. Ju K. O. Song M. S. Lee K. S. (2016). Effects of smartphone overuse on perceived cognitive function, fatigue, and daytime sleepiness among college students. J. Kor. Soc. Sch. Health 29, 245–255. doi: 10.15434/KSSH.2016.29.3.245

[ref30] Jung B. H. Kim H. K. (2016). Self-disclosure on Mobile instant messenger: the structure relationships among self-esteem, loneliness and motives for using KakaoTalk. Product. Rev. 30, 169–196. doi: 10.15843/kpapr.30.2.201606.169

[ref31] Kim J. H. Lau C. H. Cheuk K. K. Kan P. Hui H. L. Griffiths S. M. (2010). Brief report: predictors of heavy internet use and associations with health-promoting and health risk behaviors among Hong Kong university students. J. Adolesc. 33, 215–220. doi: 10.1016/j.adolescence.2009.03.012 19427030

[ref32] Kraut R. Patterson M. Lundmark V. Kiesler S. Mukophadhyay T. Scherlis W. (1998). Internet paradox: a social technology that reduces social involvement and psychological well-being? Am. Psychol. 53:1017. doi: 10.1037/0003-066X.53.9.1017 9841579

[ref33] Lam S. S. M. Jivraj S. Scholes S. (2020). Exploring the relationship between internet use and mental health among older adults in England: longitudinal observational study. J. Med. Internet Res. 22:e15683. doi: 10.2196/15683 32718913 PMC7420689

[ref34] LaRose R. Eastin M. S. Gregg J. (2001). Reformulating the internet paradox: Social cognitive explanations of internet use and depression.[on-line]. Dostupné Z. Available at: www.behavior.net/JOB/v1n2/paradox.html

[ref35] Liao S. Zhou Y. Liu Y. Wang R. (2020). Variety, frequency, and type of internet use and its association with risk of depression in middle-and older-aged Chinese: a cross-sectional study. J. Affect. Disord. 273, 280–290. doi: 10.1016/j.jad.2020.04.022 32421614

[ref36] Lind J. T. Mehlum H. (2010). With or without U? The appropriate test for a U-shaped relationship. Oxf. Bull. Econ. Stat. 72, 109–118. doi: 10.1111/j.1468-0084.2009.00569.x

[ref37] Liu Y. Li H. Hu F. (2013). Website attributes in urging online impulse purchase: an empirical investigation on consumer perceptions. Decis. Support. Syst. 55, 829–837. doi: 10.1016/j.dss.2013.04.001

[ref38] Lyu S. Sun J. (2021). Internet use and self-rated health among Chinese older adults: the mediating role of social capital. Geriatr Gerontol Int 21, 34–38. doi: 10.1111/ggi.14090 33280230

[ref39] MacKinnon D. P. Krull J. L. Lockwood C. M. (2000). Equivalence of the mediation, confounding and suppression effect. Prev. Sci. 1, 173–181. doi: 10.1023/A:1026595011371 11523746 PMC2819361

[ref40] Miao P. (2022). Accelerating the construction of the Elderly’s public psychological services system in the era of longevity. Administr. Reform 6, 94–99. doi: 10.14150/j.cnki.1674-7453.2022.06.011

[ref41] Noel J. G. Epstein J. (2003). Social support and health among senior internet users: results of an online survey. J. Technol. Hum. Serv. 21, 35–54. doi: 10.1300/J017v21n03_03

[ref42] Preacher K. J. Hayes A. F. (2008). Asymptotic and resampling strategies for assessing and comparing indirect effects in multiple mediator models. Behav. Res. Methods 40, 879–891. doi: 10.3758/BRM.40.3.879 18697684

[ref43] Preacher K. J. Rucker D. D. Hayes A. F. (2007). Addressing moderated mediation hypotheses: theory, methods, and prescriptions. Multivar. Behav. Res. 42, 185–227. doi: 10.1080/00273170701341316 26821081

[ref44] Rosenbaum P. R. Rubin D. B. (1985). Constructing a control group using multivariate matched sampling methods that incorporate the propensity score. Am. Stat. 39, 33–38. doi: 10.1080/00031305.1985.10479383

[ref45] Sabatini F. Sarracino F. (2017). Online networks and subjective well-being. Kyklos 70, 456–480. doi: 10.1111/kykl.12145

[ref46] Sasabuchi S. (1980). A test of a multivariate normal mean with composite hypotheses determined by linear inequalities. Biometrika 67, 429–439. doi: 10.1093/biomet/67.2.429

[ref47] Saxena S. Setoya Y. (2014). World Health Organization’s comprehensive mental health action plan 2013-2020. Psychiatry Clin. Neurosci. 68:585. doi: 10.1111/pcn.12207 25070129

[ref48] Schiffrin A. (2015). The press and the financial crisis: a review of the literature. Sociol. Compass 9, 639–653. doi: 10.1111/soc4.12288

[ref49] Schücker L. Hagemann N. Strauss B. (2013). Attentional processes and choking under pressure. Percept. Mot. Skills 116, 671–689. doi: 10.2466/30.25.PMS.116.2.671-689 24032339

[ref50] Senik C. (2011). Is happiness different from flourishing? Cross-country evidence from the ESS. Rev. Econom. Politique 121, 17–34. doi: 10.2307/43859900

[ref51] Sharit J. Hernández M. A. Czaja S. J. Pirolli P. (2008). Investigating the roles of knowledge and cognitive abilities in older adult information seeking on the web. ACM Trans. Comput. Hum. Interact. 15, 1–25. doi: 10.1145/1352782.1352785 PMC2792941 20011130

[ref52] Shaw L. H. Gant L. M. (2004). In defense of the internet: the relationship between internet communication and depression, loneliness, self-esteem, and perceived social support. Rev. Psicol. Trabajo Organiz. 37, 157–171. doi: 10.1089/109493102753770552 12025883

[ref53] Strauss G. P. Allen D. N. (2009). Positive and negative emotions uniquely capture attention. Appl. Neuropsychol. 16, 144–149. doi: 10.1080/09084280802636413 19430997

[ref54] Sum S. Mathews R. Hughes I. (2009). Participation of older adults in cyberspace: how Australian older adults use the internet. Australas. J. Ageing 28:189. doi: 10.1111/j.1741-6612.2009.00374.x 19951340

[ref55] Thompson G. Foth D. (2005). Cognitive-training programs for older adults: what are they and can they enhance mental fitness? Educ. Gerontol. 31, 603–626. doi: 10.1080/03601270591003364

[ref56] van Boekel L. C. Peek S. T. Luijkx K. G. (2017). Diversity in older adults’ use of the internet: identifying subgroups through latent class analysis. J. Med. Internet Res. 19:e6853. doi: 10.2196/jmir.6853 PMC5463053 28539302

[ref57] Weiser E. B. (2001). The functions of internet use and their social, psychological, and interpersonal consequences. CyberPsychol. Behav. 4, 723–743. doi: 10.1089/109493101753376678 11800180

[ref58] Wen Z. Ye B. (2014). Analyses of mediating effects: the development of methods and models. Adv. Psychol. Sci. 22:731. doi: 10.3724/SP.J.1042.2014.00731

[ref59] Wilmer H. H. Sherman L. E. Chein J. M. (2017). Smartphones and cognition: a review of research exploring the links between mobile technology habits and cognitive functioning. Front. Psychol. 8:605. doi: 10.3389/fpsyg.2017.00605 28487665 PMC5403814

[ref60] Xie Y. Hu J. (2015). An introduction to the China family panel studies (CFPS). Chin. Sociol. Rev. 47, 3–29. doi: 10.2753/CSA2162-0555470101

[ref61] Xie Y. Lu P. (2015). The sampling design of the China family panel studies (CFPS). Chin. J. Sociol. 1, 471–484. doi: 10.1177/2057150X15614535 29854418 PMC5973535

[ref62] Xiong J. Zuo M. (2019). How does family support work when older adults obtain information from mobile internet? Inf. Technol. People 32, 1496–1516. doi: 10.1108/ITP-02-2018-0060

[ref63] Xu Y. Lai D. (2021). Internet use, risk perception and urban residents. Health. J. Party Sch. Cent. Commun. 25, 100–110. doi: 10.14119/j.cnki.zgxb.2021.01.009

[ref64] Yang H. L. Zhang S. Zhang S. Q. Xie L. Wu Y. Y. Yao Y. D.. (2021). Internet use and depressive symptoms among older adults in China. Front. Psychol. 12: 739085. doi: 10.3389/fpsyt.2021.739085 PMC8688754 34950065

[ref65] Young K. S. (2009). Internet addiction: the emergence of a new clinical disorder. CyberPsychol. Behav. 1, 237–244. doi: 10.1089/cpb.1998.1.237

[ref66] Young K. Pistner M. O’mara J. Buchanan J. (1999). Cyber disorders: the mental health concern for the new millennium. CyberPsychol. Behav. 2, 475–479. doi: 10.1089/cpb.1999.2.475 19178220

[ref67] Zhang N. (2022). Risk perception, mental health distress, and flourishing during the COVID-19 pandemic in China: the role of positive and negative affect. Curr. Psychol. 1-9. doi: 10.1007/s12144-021-02624-4 PMC8754583 35039736

[ref68] Zhang S. Zhang Y. (2021). The relationship between internet use and mental health among older adults in China: the mediating role of physical exercise. Risk Manag. Healthcare Policy 14, 4697–4708. doi: 10.2147/RMHP.S338183 PMC8633706 34866945

[ref69] Zhao J. Liu Z. (2020). The impact of internet use on the health of the elderly. Chin. J. Popul. Sci. 5, 14–26, 126.

[ref70] Zhao X. Lynch J. G. Chen Q. (2010). Reconsidering baron and Kenny: myths and truths about mediation analysis. J. Consum. Res. 37, 197–206. doi: 10.1086/651257

[ref71] Zhaoran X. An’an H. Lihua H. (2017). A review of literature on the Elderly’s internet usage behavior. Libr. Inform. Serv. 61:140. doi: 10.13266/j.issn.0252-3116.2017.20.015

[ref72] Zheng Y. Zhou Z. Liu Q. Yang X. Fan C. (2019). Perceived stress and life satisfaction: a multiple mediation model of self-control and rumination. J. Child Fam. Stud. 28, 3091–3097. doi: 10.1007/s10826-019-01486-6

[ref73] Zhu Y. Zhou Y. Long C. Yi C. (2021). The relationship between internet use and health among older adults in China: the mediating role of social capital. Health 9:559. doi: 10.3390/healthcare9050559 PMC8151524 34068702

